# Laparoscopic training should be equitable for all: the impact of a mandatory, cost-neutral simulation training programme incorporating a free take-home box trainer

**DOI:** 10.52054/FVVO.16.4.045

**Published:** 2024-12-27

**Authors:** N Harvey, L Beard, N Abdulkader, C Goumalatsou, M Adamczyk, R Mallick

**Affiliations:** University Hospitals Sussex NHS Foundation Trust, Brighton, UK; Barts Health NHS Foundation Trust, London, UK; Ashford St Peter’s Hospitals NHS Foundation Trust, Woking, UK; * Joint first authors

## Abstract

**Background:**

The quality of gynaecological surgical training has faced mounting criticism internationally with multiple countries publishing potential remedies for improvement. Simulation has the indisputable ability to mitigate against training deficiencies, however, access to and the quality of simulation varies across regions, never mind nations.

**Objectives:**

To assess the effect on surgical skills by the introduction of a structured and integrated simulation programme with the unique aspect of being completely free of cost with the provision of a take-home laparoscopy box trainer (LBT).

**Materials and Methods:**

The course was mandatory in attendance and was divided into basic, intermediate and advanced streams. Each stream had a bespoke curriculum based on RCOG training. It was delivered through a combination of lectures and a mixture of dry/wet lab training sessions with the LBT provided for home use.

**Main outcome measures:**

All participants completed a pre- and post-course questionnaire with objective laparoscopic skill metrics assessed using the Inovus LapAR system at the beginning and end of the course.

**Results:**

100% of trainees demonstrated a statistically significant (p=<0.05) improvement in smoothness, time and speed. Furthermore, 100% reported the course improved their surgical skills which were further developed by LBT practice.

**Conclusion:**

This demonstrated improvement in surgical skills and confidence solidifies the hope that such a programme could be implemented as an international gynaecological standard. If implemented from the initial specialist years of training, a strong foundation can be instilled to ensure that each future gynaecologist has strong surgical skills built from a high level of laparoscopic simulation.

**What is new?:**

Our study is the first of its kind to describe an equitable and fair approach to laparoscopic surgery training; for the many rather than the select few.

## Introduction

For many years, there have been real concerns in the UK regarding the quality of gynaecological surgical training with many Royal College of Obstetricians and Gynaecologists (RCOG) trainees failing to meet basic, intermediate and advanced surgical training competencies. However, it must be stated these challenges are not unique to RCOG trainees, with the quality of gynaecological surgical training facing scrutiny throughout the globe. Concerns have been published by gynaecologists in nations such as Australia, New Zealand, Belgium, Republic of Ireland and Canada to name a small few (Obermair et al., 2009; Scheele et al., 2014; Galvin et al., 2023, Jamal et al, 2014).

The RCOG 2021 Training Evaluation Form (TEF) reported the lack of training was felt more in gynaecology training than in obstetrics with trainees experiencing insufficient theatre opportunities to fulfil their gynaecology training requirements, especially at the basic and intermediate training levels (RCOG, 2021). Furthermore, changes in the curriculum have resulted in laparoscopic salpingectomy being the most advanced surgical procedure that UK trainees are required to do prior to completion of training. Therefore, it is not a surprise that many new UK consultants report feeling underconfident in surgical procedures (BSGE, 2021). These challenges were further exacerbated by the COVID–19 pandemic (Duggan et al, 2022). Trainee surveys undertaken during the pandemic and in the recovery, period highlight the loss of surgical training opportunities, the reduction in theatre exposure and concerns surrounding the development of gynaecological surgical skills (Mallick et al., 2021; Hablase et al., 2022; Boekhorst et al., 2021).

Simulation training has the potential to alleviate training concerns, and it provides the cornerstone of safe obstetrics training with many aspects of maternity clinical best practice engrained in simulation. With regards to surgical skills, there is a wide body of evidence across many surgical specialties highlighting the wide benefits of simulation including improved hand-eye coordination and psychomotor skills, better uptake, retention and maintenance of practical skills and increased trainer and trainee confidence (Zendejas et al., 2013; Humm et al., 2022; Papanikolaou et al., 2019; Zimmerman et al., 2021).

To address trainee concerns, a gynaecology laparoscopy simulation training programme was established during the COVID-19 pandemic (December 2021) in the south of England. The programme is described in this paper, where it combined theoretical knowledge linked to the RCOG core and advanced curriculums. Practical sessions allowed trainees to practice and develop skills with faculty supervision. The provision of a laparoscopic box trainer and training package enabled each trainee to practice and consolidate their skills at home. To our knowledge is the first fully structured and funded training programme to be implemented in the UK, with the free provision of a laparoscopic box trainer and training package.

## Methods

The course was mandatory for all trainees in the region to attend and each trainee was given their own personal box trainer and package. It was divided into 3 streams; basic, intermediate and advanced and each had a bespoke simulation curriculum, relevant to the stage of RCOG training delivered through a combination of theoretical lectures and a mixture of dry/wet lab training sessions. The training programme was conceived by 5 core faculty members and was supported by over 40 other faculty members made up of gynaecology consultants and senior trainees.

The face-to-face sessions were based at a central hub in the region and the training programme was divided into three streams accommodating a maximum of 20 delegates per stream, based on their current training level. No other delegate demographics were collected. The course curriculum was matched to the RCOG training matrix and delivered over a 12-month period (Appendix 1).

The theoretical lecture-based learning covered core surgical principles such as theatre set-up, ergonomics, anatomy, electrosurgery, management of gynaecological emergencies, step-by-step laparoscopic hysterectomy and neuropelveology. Tasks included performing laparoscopic salpingectomies, ovarian cystectomy, suturing and myomectomies using either laparoscopic simulated programmes or animal or wet tissue models. The practical sessions included multiple hand-eye coordination exercises, suturing, low-and high-fidelity models, as well as animal tissues and electrosurgical equipment. Attendees were recommended to consolidate their learning at home using their box trainers.

All participants completed an anonymised non-validated questionnaire on laparoscopic knowledge, skills and trainee surgical confidence before their first session and after their final session using a 10-point numeric rating scale and a linear numeric scale.

They also performed stratified laparoscopic pass exercises utilising the Inovus LapAR system (www.inovus.org) at the beginning and end of the course. LapAR is a hybrid high- fidelity laparoscopic simulator which combines augmented reality technology with a box trainer model. It can track instrument handling and performance metrics with performance data displayed in their ‘Totum’ platform. The ‘Totum’ platform maps instrument motions and records them digitally, generating objective data on surgical performance which is shared with the facilitators. Trainees were given a simple manipulation and grasping task that was conducted and analysed using four key aspects: time; speed; smoothness and distance travelled.

In the initial stages of development in 2021, funding was agreed at a regional level. This budget covered the costs of a box trainer for each individual to keep, as well as course fees. Each box trainer contained instruments (Johan and Maryland graspers, scissors and needle holders) and a supply of training exercises and required a laptop or computer screen. Ethical approval was not required as per HRA advice as the aim of the course was trainee education.

Data was considered to be continuous (both a 10-point numeric rating scale and variables time, speed, smoothness and distance travelled) and were analysed using SPSS version 29.0 using paired t-tests. A p-value of <0.05 was considered to be statistically significant. Thematic analysis was also undertaken of trainees’ comments.

Furthermore, a questionnaire was sent to trainees at the 12-month mark of course completion to ascertain the lasting effects of the course itself in the operating theatre and the effect of an at-home laparoscopic box trainer.

## Results

### Laparoscopic pass exercises

**Figure 1:**
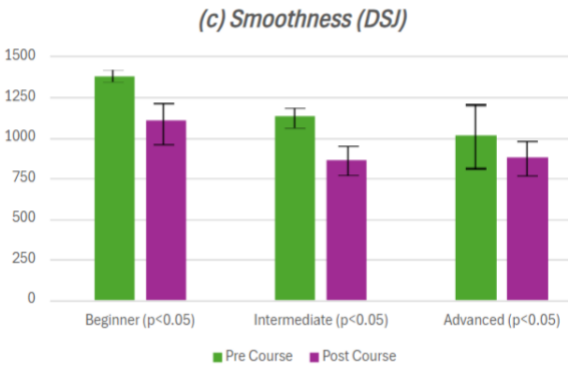
Data set comparing pre- and post-course responses of beginner, intermediate and advanced streams in (a) Speed (b) Time (c) Smoothness and (d) Distance.

There was a statistically significant improvement across time, speed and smoothness between the first and the last session of the course for the basic beginner stream (Figure 1/ Table I). There was no statistical significance when comparing distance (P=0.190) (Figure 1).

**Table I:** Pre- and post-course Lap AR exercises for each stream.

Laparoscopic skill analysed	Stream	Pre course (mean)	Post course (mean)	P-value
Time	Beginner	9.9	6.03	<0.05
	Intermediate	8.06	4.6	<0.05
	Advanced	5.8	4.2	<0.05
Speed	Beginner	0.21	0.36	<0.05
	Intermediate	0.29	0.35	<0.05
	Advanced	0.32	0.38	<0.05
Smoothness	Beginner	1376.47	1105.88	<0.05
	Intermediate	1135	857	<0.05
	Advanced	1010	880	<0.05
Distance travelled	Beginner	63.97	57.63	0.190
	Intermediate	51.5	50.3	0.677
	Advanced	48.2	42.7	0.163

In the intermediate stream, there was a statistically significant improvement in time; speed and smoothness between the first and the last session of the course (Figure 1/ Table I). There was no statistical significance when comparing distance (P=0.677) (Figure 1).

In the advanced cohort, there was a statistically significant improvement across time; speed and smoothness between the first and the last session of the course (Figure 1/Table I). There was no statistical significance when comparing distance (P=0.163) (Figure 1).

### Skills, confidence and knowledge

In the basic beginner stream, there was a statistical significance noted in all parameters of the survey pre- and post-course with participants feeling more confident in all parameters post-course (Table II). The questionnaire revealed that 100% of trainees found the laparoscopic box trainer helpful/ extremely helpful and 100% of trainees agreed/ strongly agreed that the course had improved their laparoscopic skills. 95% agreed/strongly agreed the course had improved their confidence in theatre and 85% agreed/strongly agreed the course had allowed them to capitalise on more real-life theatre opportunities. Of the participants, 100% would recommend the course to a colleague.

**Table II:** Data sets for each stream comparing the initial survey response to the post-course survey response.

Survey Questions	Beginner P Value	Intermediate P Value	Advanced P Value
How would you rate your current knowledge of pelvic anatomy?	<.05	.251	.479
How would you rate your current knowledge of the laparoscopic stack and equipment?	<.05	.066	.193
How would you rate your current knowledge of laparoscopic entry techniques?	<.05	<.05	.555
How would you rate your current knowledge of electrosurgery?	<.05	<.05	.111
How would you rate your current knowledge of the surgical management of ectopic pregnancies?	<.05	<.05	.496
How would you rate your laparoscopic skills overall?	<.05	<.05	<0.05
How would you rate your hand-eye-coordination?	<.05	<.05	.053
How confident are you that you could perform a safe laparoscopic entry?	<.05	.061	1.000
How confident are you that you could perform a basic diagnostic laparoscopy?	<.05	<.05	.443
How confident are you that you could perform a laparoscopic salpingectomy?	<.05	<.05	.591
How would you rate your current knowledge of surgical complications?	<.05	.059	.168
How confident are you that you could perform a laparoscopic oophorectomy?		<.05	.244
How confident are you that you could perform a laparoscopic ovarian cystectomy?		<.05	.070
How confident are you that you could perform a laparoscopic hysterectomy?		<.05	<0.05
How confident are you with laparoscopic suturing skills?			<0.05
How confident are you that you could perform a ureterolysis?			<0.05
How confident are you that you could perform a laparoscopic myomectomy?			<0.05

Whilst there was no statistical significance in terms of pre- and post-course survey responses for baseline knowledge in pelvic anatomy and laparoscopic stack set-up for the intermediate group, a statistical significance was noted with other topics such as electrosurgery and surgical complications and the more advanced procedural skills such as laparoscopic suturing and laparoscopic hysterectomy (Table II) with candidates expressing more confidence whilst conducting these procedures. The questionnaire revealed that 100% of trainees found the laparoscopic box trainer helpful/extremely helpful and 100% of trainees agreed/strongly agreed that the course had improved their laparoscopic skills. 100% agreed/strongly agreed the course had improved their confidence in theatre and 82% agreed/strongly agreed the course had allowed them to capitalise on more real-life theatre opportunities. 100% would recommend the course to a colleague.

The advanced group showed a statistical significance in confidence levels when performing more complex procedures such as laparoscopic myomectomy and when rating their overall laparoscopic skills (Table II). The questionnaire revealed 100% of trainees found the laparoscopic box trainer helpful/extremely helpful and 100% of trainees agreed/strongly agreed that the course had improved their laparoscopic skills. 100% agreed/ strongly agreed the course had improved their confidence in theatre and 82% agreed/strongly agreed the course had allowed them to capitalise on more real-life theatre opportunities. 100% would recommend the course to a colleague.

### Follow up questionnaire 12 months after completion of the course

A follow up questionnaire was sent to participants after 12 months of completion of the course. Of the 58 responses, 41.4% were from the beginner stream, 36.2% from the intermediate and 32.8% from the advanced group.

93.1% of attendees reported that attending the laparoscopy course improved their confidence surgically in the 12 months with 82.7% reporting a lasting improvement in surgical instruments, clinical anatomy and procedures.

The most positive feedback was regarding providing a take-home laparoscopic box trainer (LBT). Encouragingly, 3.4% of trainees reported using it daily, whilst 31% of trainees reported using it weekly and 44.8% reporting monthly use. 98.3% feel the LBT allows ongoing development of their laparoscopy skills and 81% are more confident in theatre as a result. 96.5% of the trainees feel a LBT should be a mandatory training provision, with 93.1% feeling it should be given at the start of the ST1 training year.

## Discussion

The quality of gynaecological surgical training has been under a harsh spotlight for many years now (Moss et al., 2011). In the UK, a majority of basic and intermediate trainees reported insufficient opportunities to fulfil mandatory gynaecology training requirements and only 50% of senior trainees undertaking gynaecology operative advanced training specialist modules (ATSMs) felt they were ready for independent practice (RCOG TEF 2019). Unfortunately, it is not surprising then that newly qualified consultants do not feel confident surgically (BSGE, 2021). These challenges are mirrored across the globe with multiple nations across Europe and beyond reporting significant challenges in adequate surgical exposure which is leading to a decline in surgical skills (Obermair et al., 2009; Scheele et al., 2014; Galvin et al., 2023, Jamal et al, 2014)

Simulation training has the potential to mitigate against these surgical training challenges and there is already a large body of evidence highlighting its benefits across several specialities. The use of simulation training has been widely demonstrated to improve psychomotor skills as well as surgical confidence in laparoscopic surgery (Zendejas et al., 2013; Humm et al., 2022; Papanikolaou et al., 2019; Zimmerman et al., 2021; Kundhal and Grantcharov, 2009) which is supported by our findings. In each trainee group (beginner, intermediate and advanced) measurable surgical skills such as time, speed and smoothness were all statistically improved. Furthermore, trainees reported increased confidence in their skills and theoretical knowledge. Interestingly, the biggest improvement in these skills was noted in the beginner group which was also the finding of Akdemir et al. (2014). This highlights that gynaecology simulation training should be embedded from the junior year of training to procure noticeable advancement in surgical skills, rather than the unofficial consensus that gynaecological surgery is to be focused on during advanced training.

Laparoscopic surgery also has a much longer learning curve than open surgery and expertise is gained through meticulous training and practice (De Win et al., 2016). The psychomotor process in learning is achieved through refining cerebellar motor pathways utilising repetitive practice (Hamid et al., 2021) and studies have confirmed repetition, and a spaced-out curriculum have better outcomes and shorten the learning curve when compared to stand-alone courses (Kumar and Gill, 2006; Joiya et al., 2021; Hamid et al., 2021; De Win et al., 2016). Furthermore, Hoopes et al. (2020) coined the term ‘surgical skill decay’ during the pandemic based on military findings where cognitive decline of well- trained skills were reported at 6 months and motor decline at 10 months. They highlighted a variety of methods, such as laparoscopic box trainers, virtual reality laparoscopic trainers and online webinars that could be utilised to maintain operative skills during times of reduced surgical exposure. These theories of repetition and skill retention as well as surgical skill decay were integral to our course design – a multi-session programme was developed rather than one-off stand-alone courses and all trainees were given a laparoscopic box trainer to facilitate ongoing training at home. Our results highlight the high trainee satisfaction with this approach; 100% of trainees attending the course found the take-home laparoscopic box trainer helpful and 100% agreed/ strongly agreed that the course had improved their laparoscopic skills. Furthermore, as highlighted by the follow-up survey, trainees continue to use the laparoscopic box trainer following the course with 3.4% of trainees using it daily, 31% of trainees using it weekly and 44.8% using it monthly. 98.3% felt having the box trainer allowed ongoing development of their laparoscopy skills and maintained confidence in the theatre environment.

Equity in surgical training is also key to safeguarding its future and whilst there are limited studies assessing barriers to accessing training and the drivers to choosing the specific subspecialties in gynaecology, potential barriers can be extrapolated from other surgical specialities and include cost, gender and ethnicity (Wilkinson et al., 2021; Gerges et al., 2023; Woolf et al., 2019; Bellini et al., 2019). It is key to tackle such potential barriers to accessing training to ensure rich diversity in the future workforce and ensure opportunities are equal for all. This simulation programme was created using these ideologies and appears to be the first of its kind developed in the UK which is free of charge to attend for all trainees ensuring true equity. This removes any concern regarding cost and levels the playing field for all trainees, as opposed to the self- selected few.

### Strengths

The main strength of our study was the overwhelmingly positive feedback from trainees. Regarding surgical skills, 100% of trainees attending the course found the take-home laparoscopic box trainer helpful and 100% agreed/ strongly agreed that the course had improved their laparoscopic skills. 95-100% agreed/strongly agreed that the course had also improved their confidence in theatre. The course was well received with 100% of attendees recommending it positively to a colleague. With the recent TEF feedback showing dwindling satisfaction in gynaecology training, such positive feedback should provide educators with an incentive to make a change.

Furthermore, our study highlights how a well- formatted, robust and integrated laparoscopic training programme can make a significant difference to surgical skills, regardless of training level. Within 12 months, each training group showed a statistically significant improvement across time; speed and smoothness between the first and the last session of the course.

Importantly, the study is easily replicable in format. It requires enthusiastic trainers with the main financial investment securing the take- home laparoscopy box. One of the authors of this paper (RM) was also actively involved in an RCOG task force to share the positive findings of the KSS laparoscopy course and helped develop the nationwide programme ‘Gynaecological laparoscopic simulation toolkit’. Published on the RCOG website, the toolkit is based on the format of our programme and allows easy replication of such a simulation programme so that it can be implemented as a national and ideally, as an international gynaecological standard.

### Limitations

We recognise the limitations of our study group in terms of size and homogenous geographical location.

### Interpretation

Our study strengthens the argument for promoting gynaecological surgical simulation to the same level of the long-revered obstetric simulation. This should be established from the start of postgraduate O&G training with a focus on advancing laparoscopic and surgical skills assimilated into the core curriculum. We advocate this not only for the UK but on an international platform to halt the chronic decline in gynaecology training and loss of surgical skills. Gynaecologists must be highly skilled consultants who can also tackle the huge discrepancy in women’s health, which has only widened following the COVID-19 pandemic.

### Recommendations

A structured laparoscopic simulation programme should be embedded from the start of gynaecological training as an international gynaecology standard. Introducing a standardised approach with the format as described here (Appendix 1) will allow all trainees to gain equitable access to simulation training and develop safe surgical skills from an early stage. This is a crucial step to safeguarding training for the future and maintaining high-quality gynaecologists.

## Conclusions

Our findings confirm that a mandatory, cost- neutral laparoscopic simulation programme from the start of gynaecological training improves trainee laparoscopic competencies and can address growing international concerns regarding declining gynaecology surgical skills. Furthermore, it has the potential to safeguard gynaecological training long term and ultimately the calibre of specialists produced. Additionally, it can potentially resolve long-reported trainee dissatisfaction regarding gynaecology.

This programme, or an equivalent, must be equitable and accessible to all trainees if established as a mandatory national or international standard. It is time for an investment in both obstetrics and gynaecology simulation so that all aspects of women’s health can be addressed by highly skilled, safe and confident clinicians.

## Appendix 1

https://qrco.de/bfcKPe
