# Supplementary material for: Laparoscopic training should be equitable for all: the impact of a mandatory, cost-neutral simulation training programme incorporating a free take-home box trainer
Source: Facts Views Vis Obgyn. 2024 Dec 27;16(4):465–71. doi: 10.52054/FVVO.16.4.045 (PMC11819793; doi:10.52054/FVVO.16.4.045)
Supplement: Supplementary file 1 [file FVVinObGyn-16-465-app.pdf]

Appendix 1

Basic/Beginner Stream

Theory:

Theatre environment

Pelvic anatomy - basic

Laparoscopic equipment

Electrosurgery

Consent

Laparoscopic entry techniques

Diagnostic laparoscopy

Dissection techniques

Ectopic pregnancy

Surgical complications

Introduction to suturing techniques

Practical exercises:

Basic hand eye co-ordination exercises

Inovus Ring stack exercise

Inovus Bead stack exercise

Inovus Shape manipulation exercise

Inovus Maze exercise

Inovus threading exercise

Sugar cube stack

Shape cutting exercise

Sweetie unwrap exercise

Diagnostic laparoscopy – veress needle entry simulation

Needle handing

Sutuing

Intra-coporeal knot tying

Electrosurgery hands on – monopolar, bipolar and advanced energy

Wet lab – tissue handling and dissection with animal tissue

Ectopic pregnancy simulation model

Intermediate Stream

Theory:

Pelvic anatomy – introduction to sidewall anatomy + pelvic nerves

Laparoscopic equipment including advanced energy devices

Electrosurgery recap

Laparoscopic entry technique recap

Consent

Ectopic pregnancy – diagnosis and surgical management including extra tubal ectopic pregnancies

Laparoscopic salpingo-oophorectomy

Ovarian pathology

Ovarian cystectomy

Anaesthetic considerations for laparoscopic surgery

Surgical complications – urinary tract

Surgical complications – bowel

Surgical complications – vessels and major haemorrhage

Tissue retrieval techniques

Suturing techniques

Practical exercises:

Basic hand eye co-ordination exercises

Inovus Ring stack exercise

Inovus Bead stack exercise

Inovus Shape manipulation exercise

Inovus Maze exercise

Inovus threading exercise

Sugar cube stack

Shape cutting exercise

Sweetie unwrap exercise

Ovarian cystectomy simulation

Laparoscopic salpingectomy simulation

Needle handing

Sutuing

Intra-coporeal knot tying

Wet lab – tissue handling and dissection with animal tissue, suturing animal tissue including bladder and bowel

Advanced Stream

Theory:

Consent

Pelvic anatomy –sidewall anatomy including pelvic spaces + pelvic nerves

Laparoscopic hysterectomy

Laparoscopic subtotal hysterectomy

Laparoscopic myomectomy

Tissue retrieval techniques including morcellation

Adnexal surgery recap

Endometriosis surgery

Pelvic abscess management

Laparoscopic urogynaecology procedures

Ureterolysis and stenting

Surgical complications – urinary tract

Surgical complications – bowel

Surgical complications – vessels and major haemorrhage

Suturing techniques

Practical exercises:

Laparoscopic suturing

Myomectyomy simulation

Morcellation simulation

Laparoscopic hysterectomy simulation

Excision of endometriosis simulation

Ovarian cystectomy simulation

Wet lab – tissue handling and dissection with animal tissue, suturing animal tissue including bladder and bowel

<u>Examples of lectures to cover</u>

Basic

The laparoscopic stack and camera skills

Instruments and ergonomics

Laparoscopic entry and port insertion – part 1

Introduction to pelvic anatomy and systematic laparoscopy

Electrosurgery – part 1

Salpingectomy and salpingostomy

Ovarian cystectomy

Oophorectomy

Anaesthetic consideration in laparoscopy

Patient considerations and consent

Tissue extraction techniques

Introduction to suturing and techniques

Introduction to surgical complications – bladder, bowel and vascular

Intermediate

Theatre Setup and ergonomics

Laparoscopic entry and port insertion – part 2

Patient considerations and consent

Retroperitoneal anatomy

Ectopic pregnancy

Ovarian pathology

Adnexal Surgery

Electrosurgery – part 2

Suturing techniques

Dissection techniques

Surgical complications

Advanced

Advanced pelvic anatomy – retroperitoneal and nuero-anatomy

Ectopic pregnancy including interstitial and scar ectopics

Suturing – needleholders, sutures, setup

Suturing – types of knots

Total laparoscopic hysterectomy – step by step

Tips for the challenging hysterectomy

Subtotal hysterectomy – step by step

Laparoscopic myomectomy

In-bag morcellation and tissue extraction

Management of PID

Endometriosis surgery – a structured approach

Ureterolysis – step by step and stenting

Surgical consent

Patient considerations and consent
